# Feminine hygiene products and black women's reproductive health: a review of health inequities, racialized marketing, and practice implications

**DOI:** 10.3389/frph.2026.1903781

**Published:** 2026-08-21

**Authors:** Wendasha Jenkins Hall, Ibriana Garvey

**Affiliations:** 1 Department of Health Promotion and Physical Education, Wellstar College of Health and Human Services, Kennesaw State University, Kennesaw, GA, United States; 2 Independent Researcher, Philadelphia, PA, United States

**Keywords:** black women, feminine hygiene products, gender, health disparities, marketing, race

## Abstract

Feminine hygiene products (FHPs), including douches, washes, and deodorants, are widely marketed as tools for maintaining vaginal cleanliness and freshness despite evidence linking some products to adverse sexual and reproductive health outcomes. Black women have historically been disproportionately targeted by FHP marketing and use certain products at higher rates, raising concerns about the intersection of vaginal hygiene practices, health inequities, and structural stigma. This review synthesizes historical, public health, clinical, and sociocultural scholarship on FHPs and Black women's reproductive health, with particular attention to the role of racialized marketing, cultural norms surrounding vaginal hygiene, and patient-provider communication. The literature suggests that FHP use cannot be understood solely as an individual health behavior but must be situated within broader historical, social, and structural contexts that shape health beliefs and practices. Greater attention to culturally responsive healthcare communication, evidence-based patient education, and regulatory oversight is needed to support vaginal health and reduce reproductive health inequities among Black women.

## Introduction

Feminine hygiene products (FHPs) occupy a central yet underexamined space in the U.S. women's health marketplace (1). From menstrual products to vaginal deodorants, these items are heavily marketed and reflect enduring norms about cleanliness, femininity, and bodily control (2, 3). Despite evidence linking certain FHPs to adverse reproductive and sexual health outcomes, products such as douches, washes, and deodorants are frequently marketed with misleading health claims and remain subject to limited regulatory oversight (4). This disconnect between consumer perceptions and clinical evidence contributes to the use of potentially harmful products. These concerns are particularly salient for Black women, who have historically been targeted by feminine hygiene marketing and experience disproportionate burdens of adverse reproductive health outcomes (2).

This review synthesizes historical, public health, clinical, and sociocultural scholarship on FHPs, vaginal hygiene practices, and Black women's reproductive health. It critically examines how race, stigma, and cultural norms have shaped the marketing, use, and regulation of FHPs, as well as patient-provider communication surrounding vaginal health. These patterns are situated within broader reproductive health inequities facing Black women, including elevated rates of bacterial vaginosis, sexually transmitted infections, and adverse birth outcomes. Rather than providing an exhaustive synthesis, this review examines how messages about vaginal hygiene have pathologized natural bodily functions, reinforced stereotypes, and contributed to health disparities. In doing so, it highlights the need for culturally responsive healthcare communication, the integration of structural competency into health professions education, and stronger regulation of FHPs to support safer, evidence-based practices. Ultimately, this review calls for reframing vaginal health as a site of empowerment rather than shame and for reforms that center women's health, dignity, and autonomy.

## Feminine hygiene products in the US women's health marketplace

Feminine hygiene products (FHPs)—also known as vaginal hygiene products—are widely available and heavily marketed across the U.S. consumer landscape. Valued at over $36 billion in 2023 and projected to reach $60 billion by 2033, this segment reflects both the demand for and ongoing health messaging around menstruation management and vaginal hygiene (1). While menstrual hygiene products and vaginal lubricants support reproductive and sexual health, others—such as douches, deodorants, and washes—are often promoted with misleading claims about odor control, pH imbalances, and infection prevention (5, 6). These messages persist despite clear clinical evidence: the vagina is a self-cleaning organ with a dynamic microbiome that can be disrupted by certain hygiene practices (7, 8).

Although understudied, the use of FHPs can have significant implications for reproductive health. Douching and other FHPs containing acidic or fragranced ingredients have been linked to vaginal irritation, mucosal damage, increased susceptibility to bacterial vaginosis (BV), sexually transmitted infections (STIs), and even preterm birth (9, 10). Ironically, products labeled as “natural” or “organic” may trigger allergic reactions, genital discomfort, or pain (11, 12). Despite these risks, many consumers perceive such products as essential to vaginal hygiene—reflecting deeply rooted societal norms about cleanliness, odor, and bodily self-presentation (13).

## Gaps in FHP regulation and marketing

Despite their widespread use and potential risks, most FHPs are lightly regulated under current U.S. Food and Drug Administration (FDA) guidelines. While menstrual products and sexual lubricants are considered medical devices with strict regulatory processes intended to protect consumer safety, the FDA classifies many FHPs as cosmetics–which are not subject to stringent pre- and post-market safety evaluations (4, 14). Accordingly, manufacturers are not required to prove the safety or efficacy of these products nor are they required to disclose potentially harmful ingredients (15). This lenient regulation poses issues considering consumers often make purchasing decisions based on brand recognition, marketing claims, and product reviews, which may undermine evidence-based guidance (16–18). As a result, brands can label products as “pH balancing,” “odor controlling,” and “detoxing” with limited legal repercussions (19–21). In turn, this marketing language reinforces the myth that the vagina is inherently unclean and in need of corrective care (3, 5). These messages pathologize normal bodily functions like discharge and menstruation and contribute to a cycle of stigma, misinformation, and unsafe product use (13). Further, the marketing of these products often aligns with societal expectations of femininity and cleanliness, promoting unrealistic body ideals for women (3, 22, 23).

## Racial disparities in FHP use

FHP use is not uniform across populations. While menstrual products remain the most used FHPs, there has been a notable rise in the use of cleansing products—such as washes, sprays, and wipes—particularly among Black women. Studies consistently show that Black women use vaginal douches, scented products, and talc-based powders at higher rates than other racial and ethnic groups (24–26). This greater exposure to phthalates, volatile organic compounds (VOCs), and other potential endocrine disruptors may contribute to elevated rates of BV, pelvic inflammatory disease (PID), ovarian cancer, preterm birth, and other adverse birth outcomes among Black women (27–29).

Accordingly, these patterns must be considered in the context of broader reproductive health disparities. Black women are disproportionately affected by conditions such as BV, fibroids, PID, STIs, and preterm birth—many of which are linked to vaginal microbiome disruption (30–32). For instance, BV affects about half of Black women compared to less than a quarter of White women and is associated with increased susceptibility to HIV and other STIs (33). Black women are also three times more likely to develop uterine fibroids and are nearly 50% more likely to experience preterm birth (32). These disparities are shaped not only by biological risk but by structural inequities, including systemic sexism and racism, limited access to culturally responsive care, and ongoing stigma around Black women's bodies (34, 35).

## Historical roots of FHP use and marketing

The historical roots of FHP use and marketing are closely tied to evolving cultural attitudes toward menstruation, cleanliness, and womanhood in the late 19th and early 20th centuries (3, 36). As industrialization and consumer culture expanded, companies like Johnson & Johnson and Kimberly-Clark began producing commercial menstrual products, including disposable sanitary napkins, tampons, and douches (37). These products emerged during a period when menstruation was increasingly medicalized and framed as a condition requiring discreet management (36, 38). Marketing campaigns reinforced these gendered norms by portraying menstruation not as a natural function, but as a hygienic crisis women were expected to manage away from the gaze of their male counterparts. To illustrate, 1920s Kotex ads emphasized secrecy and self-control, presenting the product as a modern solution for the “refined” woman to avoid embarrassment and maintain respectability (23).

Accordingly, this messaging aligned with Victorian ideals that equated femininity with purity, modesty, and moral superiority—values leveraged by advertisers to frame “feminine” hygiene as a woman's duty to remain desirable and respectable (3). The rise of FHPs like douches further entrenched these notions, suggesting women's bodies required constant regulation to meet societal standards of womanhood (36, 38). By the mid-20th century, advertising established a powerful cultural script: to be a proper woman was to be discreet, odor-free, and in control of one's reproductive functions (2, 3). These themes persist in contemporary marketing, where products are still pitched as tools for managing perceived flaws in vaginal functioning, reinforcing the idea that menstruation and vaginal health must be hidden or corrected (5, 6, 13).

## Historical roots of racialized FHP use & marketing

For Black women, FHP use is not only a gendered experience but one that is also shaped by race. This intersectional dynamic is rooted in a long history of racialized and sexualized olfactory discrimination that linked Black women's bodies to notions of hypersexuality, deviance, and uncleanliness. Scholars have long shown how scent—real or perceived—has served as a tool of moral judgment (39–41). In medical and cultural discourse, vaginal odor was often linked to promiscuity or deviance, while the absence of odor signified virtue (2, 42). These associations were harmful in a society where Black women faced systemic sexual violence, including rape, forced reproduction, and nonconsensual medical experimentation (34, 43–45). This stigma imposed a dual burden: deodorize not only to appear clean but also to assert control in a context where bodily autonomy was routinely denied (2, 46, 47). Accordingly, this imperative fueled widespread use of deodorants and douches among Black women as a means of achieving an “acceptably clean” body. Consequently, hyper-cleanliness became a symbol of respectability and resistance for Black women (2, 40, 41). Even Black cultural and social institutions promoted hygiene as a defense against racist and sexist assumptions about Black women's bodies, linking cleanliness to moral worth and social protection (2, 47).

Subsequently, feminine hygiene brands exploited these anxieties in marketing to Black women. While mainstream advertisements hinted at douching as contraception for White women, campaigns in the Black press framed products like *Lysol* and *Sani-Tabs* as protection against disease and odor (2, 3, 48). Even amid cultural shifts such as the *Black Power* and *Black is Beautiful* movements, vaginal deodorants remained popular in Black media—they were presented as symbols of femininity and desirability. These products became household staples, with hygiene routines and beliefs passed down through generations and familial associations and reinforced by cultural expectations around cleanliness, morality, and femininity (2, 47, 48). Despite mounting evidence of the health risks associated with FHPs, targeted marketing to Black women has continued well into the 21st century (24, 25, 49, 50).

## Patient-provider communication about sexual health and hygiene

Women's experiences discussing sexual and reproductive health with providers are often shaped by stigma, provider discomfort, and gendered assumptions; thus, creating barriers to effective care. Patients report that providers rarely initiate these conversations unless prompted by symptoms of infection or disease (51, 52). And when addressed, discussions are typically clinical and impersonal— often overlooking the social and cultural dimensions of sexuality (53). This can leave patients feeling judged or dismissed, especially when discussing topics like vaginal odor or hygiene (54, 55). Accordingly, many turn to media, family, or product marketing for guidance which can result in provider avoidance and self-medicalization (2, 12, 47).

Conversely, providers often feel unequipped to discuss sexual health, with some citing limited training and uncertainty about appropriate language or how to address complex issues like trauma, dysfunction, or diverse sexual practices (56, 57). This discomfort may be compounded by personal beliefs, cultural norms, or fear of legal consequences, particularly when addressing sensitive topics such as sexual orientation, gender identity, or non-monogamy (58, 59). As a result, providers may avoid the topic or frame it narrowly around reproduction or disease prevention, rather than exploring broader aspects of sexual wellbeing. This avoidance can leave patients feeling stigmatized or unable to fully disclose their concerns, reinforcing a cycle of silence and misinformation (60).

Undergirding these patient-provider interactions is the critical concept of *cultural health capital* (CHC)—the knowledge, communication styles, and behaviors that patients use to navigate healthcare settings (60). CHC affects how patients communicate their needs and concerns and how providers perceive and respond to them. Research shows that patients who can speak the “language” of medicine, articulate their symptoms in ways that align with biomedical expectations, or demonstrate familiarity with healthcare norms are often viewed as more competent or credible (60, 61). In turn, providers often respond more favorably to patients who exhibit CHC that aligns with dominant norms, such as using medical language, appearing deferential, or expressing health concerns in a “rational” and unemotional way (60, 62, 63). However, Black women and other marginalized groups may engage differently, or possess forms of CHC that providers do not recognize. Instead, they may have varying forms of CHC that reflect their own lived experiences, cultural knowledge, or ways of expressing concern about their bodies that are frequently undervalued or misinterpreted by providers (60, 64). This mismatch can result in miscommunication, perceived noncompliance, or judgment, further discouraging open dialogue about sexual health.

## Practice implications for women's health professionals

### Prioritizing patient-provider conversations about vaginal hygiene practices

Silence and shame around vaginal hygiene presents challenges in clinical settings as women are vulnerable to *stereotype threat*—a disruptive psychological state triggered by the fear of confirming a negative stereotype about one's social identities (e.g., race, gender, class, or sexual orientation) (65). Stereotype threat, especially among Black women, contributes to biased clinical encounters often leading to miscommunication, underestimation of symptoms, or inadequate treatment recommendations (65, 66). Thus, prioritizing and normalizing conversations about vaginal hygiene between providers and patients is essential for improving women's health outcomes and addressing persistent health inequities (52). Open, non-judgmental dialogue allows providers to identify and address harmful practices often rooted in misinformation shaped by sociocultural norms. Also, intentional and purposeful patient-provider communication offers an opportunity to help patients (re)build trust with healthcare systems and foster culturally responsive care (67).

Importantly, demonstrating cultural humility in these conversations lays the foundation for a more inclusive model of care that enhances patients' cultural health capital (CHC) (60). Acknowledging the cultural and social dimensions of reproductive health is essential for equitable, patient-centered care—particularly for Black women and other marginalized groups—as these dimensions shape how CHC is expressed and interpreted in clinical encounters (60, 66, 68–70). When providers recognize and value diverse forms of CHC, they can counter bias, address structural barriers, enhance health literacy, and counteract the spread of health misinformation (60, 71, 72). Further, the integration of these conversations into well-woman visits promotes early intervention, preventive care, and patient empowerment—ultimately leading to better health outcomes (52). Prioritizing these conversations is a critical step toward equitable, patient-centered care that affirms women's dignity and autonomy.

### Pre-service and continuing education opportunities

Pre-service, undergraduate, and graduate medical and nursing training are pivotal for shaping healthcare providers' ability to deliver culturally responsive, equitable care—particularly in the realm of women's reproductive health (73, 74). Pre-service nursing and medical education lay the foundation for clinical practice, making it a crucial period to introduce students to the ways in which race, gender, history, and culture intersect in healthcare delivery (69, 70, 75–78). Thus, foundational coursework on the social determinants of health and cultural competence must move beyond superficial coverage. Instead, it must critically examine how systemic racism and sexism, historical trauma, and cultural backgrounds influence reproductive health practices and outcomes—especially among Black women (45, 68, 73, 74, 77, 78). If students are not adequately trained to understand these behaviors in their cultural and historical context, they risk perpetuating bias and missing critical opportunities for patient education and harm reduction (66, 79).

Building on the foundational skills developed in pre-service training, providers must recognize that the ability to deliver equitable, patient-centered care requires ongoing education well beyond initial licensure. As providers transition into graduate education and independent practice, continuing education becomes essential for deepening their knowledge and skills (80–84). Practicing clinicians must continue refining their awareness of the structural determinants of health and wellness, confronting implicit biases, and learning how to engage in sensitive conversations with empathy and transparency (85–88). Further, ongoing professional development should be supported institutionally and center cultural humility, anti-racism, and reproductive justice frameworks (87, 89, 90). Career-long learning in these areas is vital not only to improve health outcomes, but to restore trust and ensure that reproductive health is seen as integral to whole-person care—particularly for Black women.

### Support policy that promotes feminine hygiene product safety regulation

Women's health professionals play a vital role in advocating for patient safety beyond the exam room (5). This includes supporting policies and research that address the risks associated with FHPs. Despite their widespread use, items like washes, douches, and wipes are not subject to the same rigorous safety testing and labeling as other health products (14, 15, 91). This regulatory gap exposes millions to potentially harmful chemicals, including endocrine disruptors, allergens, and carcinogens (5, 24, 92, 93). Thus, advancing research and policy in this area is essential for informed consumer choices and reproductive health equity.

One notable U.S. legislative proposal is the Robin Danielson Menstrual Product and Intimate Care Product Safety Act (H.R. 5957)—a bill that has been introduced repeatedly since 1997 but has yet to gain sufficient congressional support to be enacted into law (94). This bill directs the National Institutes of Health to research the health impacts of ingredients in menstrual and intimate care products, including contaminants and substances used as fragrances, dyes, and preservatives (94, 95). Given the bill's failure to pass in Congress, health professionals and professional organizations can use their collective authority to build congressional support for the bill and press for its enactment into law.

Professional organizations like the American College of Obstetricians and Gynecologists (ACOG), Association of Women's Health, Obstetric and Neonatal Nurses (AWHONN), and the American Medical Women's Association (AMWA) also have powerful platforms to influence public awareness and policy. To date, these organizations have published several expert reviews, committee opinions, and advocacy resources regarding women's reproductive health that guide patient education, clinical practice, and health policy (96–101). Although these organizations have not specifically addressed FHPs and their potential health risks, by endorsing legislation like H.R. 5957 and advocating for expanded research funding, these groups can help shift the conversation toward transparency and safety. Their leadership would send a strong message to lawmakers, regulators, and the public about the urgency of protecting menstrual and intimate care health. Through collective advocacy, the healthcare community can help ensure safer, evidence-based products for all.

## Conclusion

Feminine hygiene products occupy a unique and often problematic space at the intersection of women's health, consumer marketing, and cultural norms. As this review demonstrates, their use—particularly among Black women—is shaped by a legacy of racialized and gendered messaging that pathologizes natural bodily functions and reinforces harmful stereotypes. Contemporary marketing continues to exploit these narratives, while regulatory gaps leave consumers vulnerable to products containing potentially harmful ingredients. The health consequences, from bacterial vaginosis to adverse birth outcomes, underscore the urgent need for change.

Addressing this issue requires a multi-pronged approach: advancing culturally responsive patient-provider communication, integrating structural competency and reproductive justice into medical education, and advocating for robust safety regulations and transparent labeling of all feminine hygiene products. For clinicians, this means moving beyond symptom management toward conversations that dismantle stigma, validate lived experiences, and promote evidence-based practices. For policymakers and professional organizations, it means elevating FHP safety as a public health priority.

Ultimately, reframing vaginal health as a site of empowerment rather than shame can disrupt cycles of misinformation, reduce preventable harm, and advance reproductive health equity. Protecting the health, dignity, and autonomy of all women demands nothing less than this coordinated effort.
